# Characterization of the Expression of the RNA Binding Protein eIF4G1 and Its Clinicopathological Correlation with Serous Ovarian Cancer

**DOI:** 10.1371/journal.pone.0163447

**Published:** 2016-09-26

**Authors:** Lanfang Li, Qingya Luo, Zhe Xie, Guiqin Li, Chengyi Mao, Yi Liu, Xin Wen, Na Yin, Jianzhong Cao, Jing Wang, Li Li, Jianhua Yu, Fang Wang, Ping Yi

**Affiliations:** 1 Department of Obstetrics and Gynecology, Research Institute of Surgery, Daping Hospital, Third Military Medical University, Chongqing, China; 2 Department of Pathology, Research Institute of Surgery, Daping Hospital, Third Military Medical University, Chongqing, China; 3 Department of Biochemistry, Institute of Basic Medical Sciences, Chinese Academy of Medical Sciences (CAMS) & Peking Union Medical College (PUMC), Beijing 100005, PR China; 4 Department of General Surgery, Peking Union Medical College Hospital, Chinese Academy of Medical Sciences & Peking Union Medical College, Beijing, China; 5 The Ohio State University of Comprehensive Cancer Center, Columbus, Oh 43210, United States of America; Sapporo Ika Daigaku, JAPAN

## Abstract

**Background:**

Ovarian cancer is the most lethal type of malignant tumor in gynecological cancers and is associated with a high percentage of late diagnosis and chemotherapy resistance. Thus, it is urgent to identify a tumor marker or a molecular target that allows early detection and effective treatment. RNA-binding proteins (RBPs) are crucial in various cellular processes at the post-transcriptional level. The eukaryotic translation initiation factor 4 gamma, 1(eIF4G1), an RNA-binding protein, facilitates the recruitment of mRNA to the ribosome, which is a rate-limiting step during the initiation phase of protein synthesis. However, little is known regarding the characteristics of eIF4G1 expression and its clinical significance in ovarian cancer. Therefore, we propose to investigate the expression and clinicopathological significance of eIF4G1 in ovarian cancer patients.

**Methods:**

We performed Real-time PCR in 40 fresh serous ovarian cancer tissues and 27 normal ovarian surface epithelial cell specimens to assess eIF4G1mRNA expression. Immunohistochemistry (IHC) was used to examine the expression of eIF4G1 at the protein level in 134 patients with serous ovarian cancer and 18 normal ovarian tissues. Statistical analysis was conducted to determine the correlation of the eIF4G1 protein levels with the clinicopathological characteristics and prognosis in ovarian cancer.

**Results:**

The expression of eIF4G1 was upregulated in serous ovarian cancer tissues at both the mRNA (P = 0.0375) and the protein (P = 0.0007) levels. The eIF4G1 expression was significantly correlated with the clinical tumor stage (P = 0.0004) and omentum metastasis (P = 0.024). Moreover, patients with low eIF4G1 protein expression had a longer overall survival time (P = 0.026).

**Conclusions:**

These data revealed that eIF4G1 is markedly expressed in serous ovarian cancer and that upregulation of the eIF4G1 protein expression is significantly associated with an advanced tumor stage. Besides, the patients with lower expression of eIF4G1 tend to have a longer overall survival time. Thus, eIF4G1 may contribute to the occurrence and metastasis of ovarian cancer and can serve as a potential therapeutic target for the treatment of ovarian cancer.

## Introduction

Ovarian cancer is the most lethal type of malignant tumor in gynecological cancers. Serous ovarian cancer is the most common histological type of epithelial ovarian cancer, accounting for 75% of epithelial ovarian cancer. It is estimated that there are nearly 52,100 newly diagnosed ovarian cancer cases and about 22,500 cancer deaths in 2015 in China [1]; moreover, ovarian cancer is the fifth leading cause of cancer-related deaths in women in the United States [2]. Because the majority of patients are diagnosed at an advanced stage and the postoperative recurrence rate is higher, there has been scarcely any change in the mortality rate of ovarian cancer since 1930 [3]. Hence, it is urgent to identify a tumor marker or a therapeutic target that allows early detection and leads to effective treatment.

RNA-binding proteins, as their name implies, are a class of proteins that can directly bind to RNA. Currently, by employing mRNA interactome capture methodology, more than 800 human RNA-binding proteins have been discovered [4]. These proteins play a critical role in determining cell fate at posttranscriptional levels, including splicing, polyadenylation, mRNA stabilization, mRNA localization, and translation. Translation is the first step of protein biosynthesis and is present in many cellular processes including cell proliferation, growth, and development. RNA-binding proteins bind to their target mRNAs and then recruit translational repressor and motor proteins that translocate the assembled messenger ribonucleoprotein particles (mRNPs) to their final destination. However, translation factors play an important role in this process. The RNA-binding protein, eIF4G1, is a subunit of the eukaryotic translation initiation complex and a necessary protein that control translation of proteins in eukaryotic cells. This protein serves as a scaffold that interacts with numerous initiation factors including PABP, eIF3, and two eIF4F components(eIF4E and RNA helicase eIF4A) to ensure the correct formation of the mRNA-ribosome complex [5]. In the past decade, eIF4G1 was identified as a novel causal gene for Parkinson’s disease (PD) by exome sequencing and genome-wide linkage analysis followed by direct sequencing [6]. Recent evidence has demonstrated that apart from its association with PD, eIF4G1 plays a crucial role in the occurrence and development of breast cancer [7], squamous cell lung carcinoma [8], malignant pleural mesothelioma [9], multiple myeloma [10] and cervical cancer [11]. However, the relation between eIF4G1 and ovarian cancer remains unclear. We designed this study to explore whether the expression of eIF4G1 influences clinicopathological features and clinical prognosis.

## Methods and Materials

### Tumor samples

We obtained 40 frozen specimens of invasive serous epithelial ovarian cancer from DAPING hospital between 2013 and 2014, snap frozen in liquid nitrogen and stored at -80°C. Twenty-seven fresh ovarian surface epithelium (OSE) brushings were obtained with a sterile cytology brush from the normal ovaries of donors during surgery for other benign gynecological diseases at DAPING Hospital between 2013 and 2015. The donors, who were approximately 50 years old, were selected because ovarian cancer most frequently strikes in this age group. We also collected some Formalin-Fixed Paraffin-Embedded (FFPE) samples from DAPING hospital between 2009 and 2016, including 134 cases of serous epithelial ovarian cancer and 18 normal ovarian tissues. Clinical and pathological information, including age, FIGO stage, omentum metastasis, differentiation, CA125, platinum-based chemotherapy sensitivity and residual tumor size, were collected from clinical records (S1 Table). None of these patients received chemoradiotherapy before surgery.

### RNA isolation and Real-time reverse transcriptase PCR

RNA was extracted from the tissues harvested using the reagent Trizol (Invitrogen, Carlsbad, CA, USA) according to the manufacturer’s instructions. The RNA was quantified by absorbance at 260 nm. Total RNA was reverse transcribed using RevertAid First Strand cDNA Synthesis Kit (Thermo Scientific,USA) according to the manufacturer's protocol, and cDNAs were subsequently analyzed by quantitative real-time PCR (qRT-PCR). Primers for eIF4G1 were as follows: forward primer: 5′-TCCAACACGTTAGTTCGAGCC-3′, reverse primer: 5′-TTCAGCACTGCAACGTCCA -3′. The data were normalized using the β-actin, whose primers were as follows: forward primer: 5′-CTGGCACCACACCTTCTACA-3′, reverse primer: 5′-AGCACAGCCTGGATAGCAAC-3′. PCR was performed in 7900HT Fast Real-Time PCR System (Applied Biosystems, USA) with SYBR Green master mix (New Industry, China). The reaction conditions were 50s at 50°C and 5 min at 95°C followed by 45 cycles of 5s at 95°C, 15s at 60°C and 10s at 72°C. The relative quantification was calculated using the ΔΔCt method and normalized based on β-actin. All samples were typically analyzed in triplicate in a minimum of three independent runs.

### Tissue array

For immunohistochemical study, FFPE samples were used to create 3 paraffin-embedded tissue microarrays. Arrays were dewaxed and then doused with endogenous peroxidase 3% hydrogen peroxide. The epitope retrieval was performed with 10 mMsodium citrate (pH 6). Nonspecific binding was blocked using PBS supplemented with 5% BSA. Primary antibody (Abcam, ab47625, 1:150) was incubated at 4°C overnight, and the appropriate secondary antibody was incubated at room temperature for 30 min. Staining for eIF4G1 intensity and frequency was scored by 2 independent investigators who were blinded to tissue type and pathological diagnosis. Immunoreactivity was scored according to the intensity of staining (-: 0; +: 1; ++: 2; and +++: 3) and the percentage of the cells of interest staining positive for each antigen (0%: 0;1%~25%: 1; 26%~50%: 2; 51%~75%:3; and >75%:4) according to the H score system. A composite score was determined by multiplying the intensity and extent scores. An optimal cutoff value was identified as the mean value of the composite score.

### Ethics Statement

Patients in this study provided written informed consent. The study of patient specimens was approved by the Institutional Review Board of DAPING Hospital at Third Military Medical University.

### Statistical analyses

Student’s *t*-test and the Mann-Whitney U Test were used to compare eIF4G1 expression in cancerous and non-cancerous tissues. The association between eIF4G1 expression and clinicopathological characteristics was analyzed using a Chi-square test. The Kaplan-Meier method and the log-rank test were applied to estimate progression-free survival (PFS), overall survival (OS), and their differences involved. Multivariate Cox regression (proportional hazard model) analysis was used to identify independent prognostic factors. Associations are calculated as hazard ratios (HR) and 95% confidence intervals (CI). All of the analyses were conducted by SPSS (Statistical Package for the Social Sciences) version 18.0 (Chicago, IL, USA). Results were considered statistically significant with a p value less than 0.05.

## Results

### Increased expression of *eIF4G1* mRNA in serous ovarian cancer

**Fig 1:**
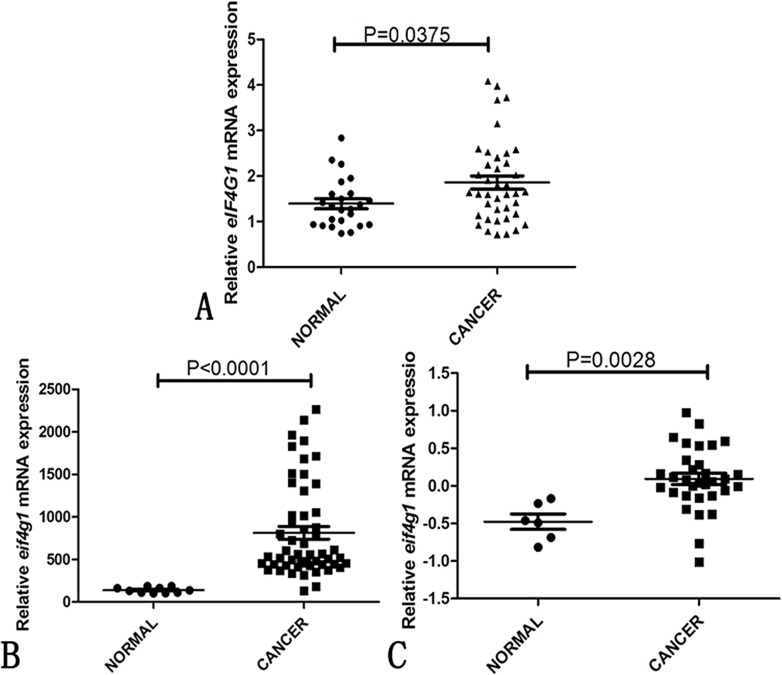
The mRNA expression of eIF4G1 in ovarian cancer tissues and normal ovary epithelial cell specimens. (A) Real-time RT PCR analysis of eIF4G1 in fresh frozen ovarian cancer samples and normal ovarian surface epithelial cell specimens in our study (P = 0.0375). (NORMAL:27 normal ovarian surface epithelial cell specimens;CANCER:40 frozen ovarian cancer samples). (B) Relative eIF4G1 mRNA expression of ovarian cancer samples and normal ovarian surface epithelial cells from the reported microarray data (accession number GSE18521) (P<0.0001; NORMAL: 10 normal ovarian tissues; CANCER: 53 snap-frozen ovarian cancer tissue specimens). (C) Relative eIF4G1 mRNA expression of ovarian cancer samples and normal ovarian surface epithelial cells from reported microarray data (accession number GSE40595; P = 0.0028; NORMAL: 6 normal ovarian surface epithelial cells; CANCER: 35 snap-frozen ovarian cancer tissue specimens). Scatter plot represents means ± SD.

We examined 40 serous ovarian cancer specimens and 27 normal OSE specimens using quantitative RT-PCR for mRNA and analyzed the difference in the eIF4G1 mRNA expression between the tumor and the normal OSE specimens. To accurately evaluate the expression levels of eIF4G1 mRNA, the included samples for RT-PCR contain at least 70% of tumor cell nuclei. The expression of eIF4G1 was markedly higher in the serous ovarian cancer specimens than in normal OSE specimens as shown in Fig 1A (P = 0.0375). Besides, we compared the *eIF4G1* mRNA expression of ovarian cancer tissues and normal ovarian surface epithelial cells from the microarray data (GEO accession numbers GSE18521 [12] and GSE40595 [13]). The expression of eIF4G1 was also higher in the serous ovarian cancer specimens (GSE18521: P<0.0001; GSE40595: P = 0.0028) as shown in Fig 1B and 1C.

### Increased expression of eIF4G1 protein in ovarian cancer

**Fig 2:**
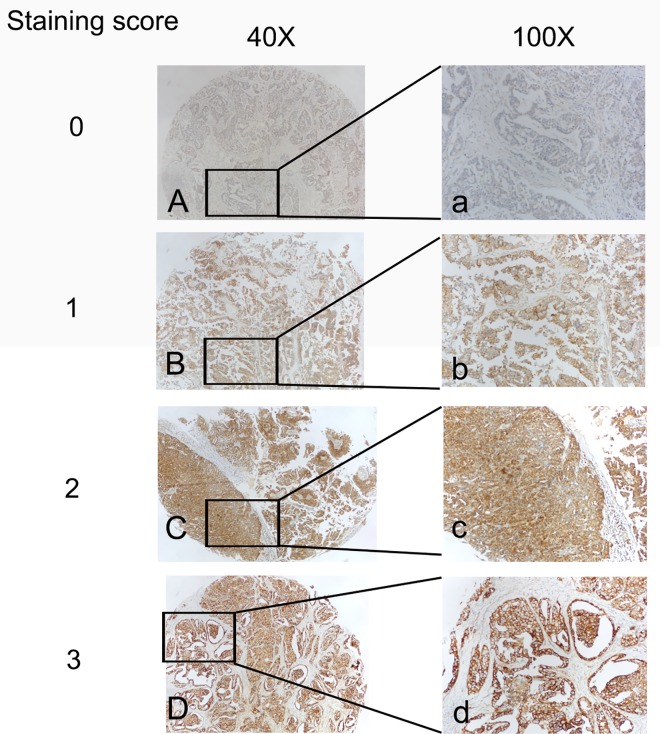
The expression of eIF4G1 at the protein level in ovarian cancer tissues from patients. **(Left, ×40; Right, ×100)** Immunohistochemical staining of eIF4G1 in ovarian cancer tissue at different staining score using anti-human eIF4G1 antibodies. (Left magnification, ×40; Right magnification, ×100).

**Fig 3:**
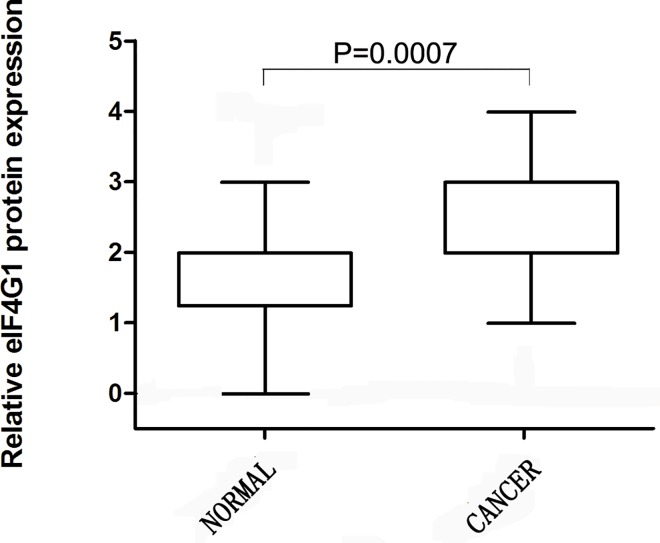
Summary of relative eIF4G1 protein expression in normal and ovarian cancer specimens assessed by IHC. Intensity of eIF4G1 staining was scored from 1 to 4, and an individual box plot was generated to display the distribution of the intensity of eIF4G1 staining for normal ovary samples and ovarian cancer specimens. Immunohistochemical analysis of eIF4G1 in FFPE ovarian cancer samples (n = 134) and normal FFPE ovarian surface epithelial specimens (n = 18). P = 0.0007. (NORMAL: normal FFPE ovarian surface epithelial specimens; CANCER: FFPE ovarian cancer samples).

To further determine whether eIF4G1 protein expression is consistent with the results for mRNA expression, we used three tissue microarrays, which contained the cores from 134 serous epithelial ovarian cancers and 18 normal ovaries to localize and quantify eIF4G1 expression. Positively stained eIF4G1 was primarily located in the cytoplasm of ovarian cancer cells and manifested as light brown and brown particles (Fig 2). In contrast to OSE, the expression of eIF4G1 was much higher in the ovarian cancer samples (P = 0.0007) (Fig 3). The histochemical score was consistent with the previous RT-PCR results for *eIF4G1*mRNA.

### Association of eIF4G1 protein expression and clinicopathological characteristics

To further investigate the possible correlations between eIF4G1 expression levels and the clinicopathological characteristics of patients, we followed 134 patients with ovarian cancer. The primary clinicopathological characteristics of ovarian cancer patients are shown in Table 1. The age of the 134 patients ranged from 31 years to 81 years (mean age, 54 years). In terms of the distribution of FIGO stage, 31 patients were at stages I and II, and 103 patients were at stages III and IV. Moreover, the majority of the patients had poorly differentiated tumors, and 61.9% of patients had undergone optimal surgical reduction of the primary tumor (residual tumor, ≤1 cm in diameter). A total of 87 patients presented positive omentum metastasis, and the remaining 46 patients showed negative metastasis. Of the patients, 46.3% had tumors that were sensitive to initial chemotherapy whereas 29.9% had refractory or resistant disease (data were unknown for 23.9% of patients who had the following conditions: refused chemotherapy, incompletion of initial chemotherapy or less than 6 months after completion of initial chemotherapy). The serum of CA-125 level of 78 patients was ≥600U/ml whereas the level of the other 54 patients was <600U/ml.

**Table 1:** Baseline Characteristics of the 134 Patients of Serous Ovarian Cancer.

Characteristics	Value
Age–yr	
Mean	54
Range	31–81
>50	81(60.4%)
= <50	53(39.6%)
Tumor stage–no.(%)	
I or II	31(23.1%)
III or IV	101(75.4%)
Differentiation–no.(%)	
Well differentiated	10(6.7%)
Poorly differentiated	124(93.3%)
Omentum metastasis–no.(%)	
Absent	46(34.3%)
Present	87(64.9%)
Missing data	1(0.8%)
Cytoreduction—no. (%)*	
Optimal	83(61.9%)
Suboptimal	51(38.1%)
Response to initial chemotherapy -no. (%)	
Sensitive	62(46.3%)
Resistant or refractory	40(29.9%)
Unknown	32(23.9%)
CA125 level (U/ml)	
<600	54(40.3%)
≥600	78(58.2%)
Missing data	2(1.5%)

* Optimal cytoreduction was defined as cytoreduction resulting in residual tumor of 1 cm or less in diameter.

**Fig 4:**
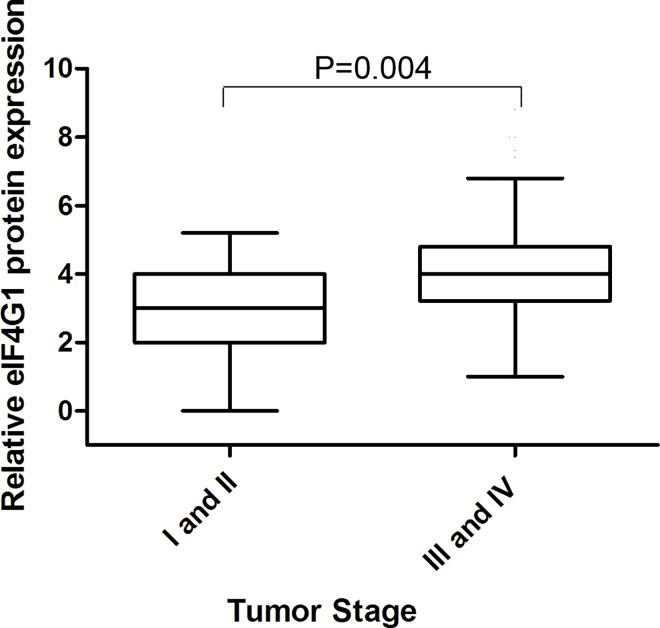
Correlation of eIF4G1 protein expression with clinicopathological stage of ovarian cancer tissues used for eIF4G1 expression analysis. Immunohistochemical analysis of eIF4G1 with stage I and II of ovarian cancer samples (n = 31) and stage III and IV of ovarian cancer samples (n = 102). P = 0.004.

**Fig 5:**
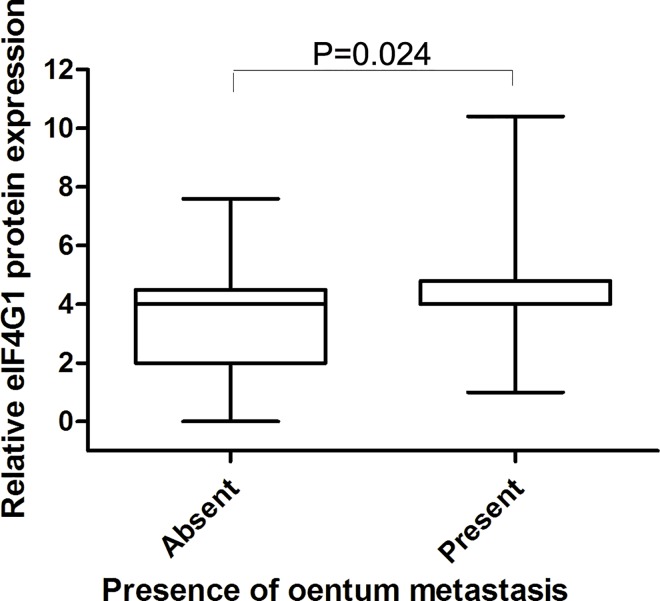
Correlation of eIF4G1 protein expression with the presence of omentum metastasis of ovarian cancer tissues used for eIF4G1 expression analysis. Immunohistochemical analysis of eIF4G1 without omentum metastasis of ovarian cancer samples (n = 46) and with omentum metastasis of ovarian cancer samples (n = 86). P = 0.024.

The mean value of staining H score in cancer tissue microarrays is 4 and the cutoff value was identified as the mean value. So a staining H score of ≥4 was used to define tumors with high eIF4G1expression, and a score of <4 indicated low eIF4G1 expression. A correlation was observed between eIF4G1 cytoplasmic expression and the pathological parameters of 134 cases of ovarian cancer (Table 2). In univariate analyses, as shown in Fig 4, lower expression of eIF4G1 protein was exhibited in early–stage ovarian cancer (FIGO stages I and II) while higher expression in advanced stage ovarian cancer (FIGO stages III and IV) tissues. (P = 0.004). In addition, a moderate but significant correlation between the level of eIF4G1 protein level and omentum metastasis was also observed (P = 0.024) (Fig 5). However, as summarized in Table 2, no remarkable correlations were detected between the expression level of eIF4G1 protein and patient age, degree of differentiation, optimal or suboptimal cytoreduction, response to chemotherapy or the serum of CA-125 in patients with ovarian cancer. In multivariate analyses, tumor stage (hazard ratio,0.349; 95% CI, 0.549 to 1.587;P = 0.012), a resistant or refractory chemoresponse (hazard ratio,8.579; 95% CI, 4.02to 18.29; P<0.0001for PFS and hazard ratio,6.76; 95% CI, 3.49 to 13.07; P<0.0001 for OS) and cytoreduction (hazard ratio,3.21; 95% CI, 1.74 to 5.92; P<0.0001 for PFS and hazard ratio,2.3; 95% CI, 1.29 to 4.08; P = 0.005 for OS) were associated with poor survival. However, after adjusting for other risk factors (age, tumor stage, chemosensitivity and cytoreduction), the eIF4G1 expression level was not an independent prognostic factor for OS (hazard ratio, 0.985; 95% CI, 0.524 to 1.85; P = 0.96) and PFS (hazard ratio, 1.235; 95% CI, 0.64 to 2.37; P = 0.53). Moreover, other clinicopathological characteristics including age, omentum and CA125 level were not independent prognostic markers for ovarian cancer (Tables 3 and 4).

**Table 2:** Associations of cancerous eIF4G1 expression with clinicopathologic characteristics of ovarian cancer.

Characteristics	eIF4G1 expression		*X*^*2*^	P-Value
	High(n%)	Low(n,%)		
Age–yr			0.263	0.608
>50	38(28.6%)	15(11.3%)		
= <50	54(40.6%)	26(19.5%)		
Tumorstage–no.(%)			8.190	0.004
I or II	15(11.3%)	16(12%)		
III or IV	77(57.9%)	25(18.8%)		
Differentiation–no.(%)			1.056	0.304
Well differentiated	5(3.7%)	5(3.7%)		
Poorly differentiated	88(65.7%)	36(26.9%)		
Omentum metastasis–no.(%)			5.084	0.024
Absent	26(19.5%)	20(15%)		
Present	65(48.9%)	21(15.8%)		
Cytoreduction—no. (%)*			3.16	0.075
Optimal	42(31.6%)	26(19.5%)		
Suboptimal	50(37.6%)	15(11.3%)		
Response to initial chemotherapy -no. (%)			0.91	0.34
Sensitive	41(30.8%)	16(12%)		
Resistant or refractory	30(22.6%)	8(6%)		
CA125 level (U/ml)			0.558	0.455
<600	36(27.1%)	18(13.5%)		
≥600	56(42.1%)	21(15.8%)		

P values were calculated after missing values were excluded.

* Optimal cytoreduction was defined as cytoreduction resulting in residual tumor of 1 cm or less in diameter.

**Table 3:** Prognostic factors of Ovarian Cancer after resection (PFS).

Variables	Number(n)	PFS(Univariate) median±SE	95%CI	P*	PFS(Multivariate) Hazard Ratio	95%CI	P^#^
Age–yr				0.495			
>50	62	21.00±4.78					
= <50	38	13.00±1.98	9.11–16.89				
Tumor stage–no.(%)				0.001	0.349	0.549–1.587	0.012
I or II	24	33.00±3.05	27.02–38.98				
III or IV	76	12.00±0.983	10.07–13.93				
Differentiation–no.(%)				0.178			
Well differentiated	9	15.00±2.73	9.66–20.34				
Poorly differentiated	91	36.00±7.78	20.63–51.37				
Omentum metastasis–no.(%)				0.001			
Absent	38	29.00±2.49	24.12–33.88				
Present	62	12.00±0.87	10.29–13.71				
Cytoreduction—no. (%)*				<0.0001	3.214	1.74–5.92	<0.0001
Optimal	69	7.00±1.53	4.01–9.99				
Suboptimal	31	26.00±2.58	20.95–31.05				
Response to initial chemotherapy -no. (%)				<0.0001	7.865	4.11–15.06	<0.0001
Sensitive	58	8.00±1.17	5.71–10.30				
Resistant or refractory	35	27.00±2.47	22.15–31.85				
CA125 level (U/ml)				0.45			
<600	43	21.00±3.64	13.87–28.13				
≥600	57	13.00±1.43	10.20–15.80				
**eIF4G1 expression**				0.187			
Low	28	26.00±9.65	7.08–44.92				
High	72	15.00±3.19	8.74–21.26				

* Log-rank test

# Cox regression test.

**Table 4:** Prognostic factors of Ovarian Cancer after resection (OS).

Variables	Number(n)	OS(Univariate) mean±SE	95%CI	P*	OS(Multivariate) Hazard Ratio	95%CI	P^#^
**Age–yr**				0.073			
>50	70	33.00±5.44	22.35–43.65				
≤50	40	24.00±1.99	20.10–27.90				
**Tumor stage–no.(%)**				<0.0001	0.373	0.165–0.844	0.018
I or II	25	50.00±2.26	45.57–54.43				
III or IV	85	23.00±1.67	19.73–26.27				
**Differentiation–no.(%)**				0.044	2.642	0.91–7.67	0.075
Well differentiated	9	58.00±11.03	36.38–79.62				
Poorly differentiated	101	25.00±1.79	21.49–28.51				
**Omentum metastasis–no.(%)**				<0.0001			
Absent	40	48.00±4.60	38.99–57.01				
Present	69	21.00±1.48	18.11–23.89				
Cytoreduction—no. (%)*				<0.0001	2.30	1.29–4.08	0.005
Optimal	71	41.00±7.53	26.24–55.76				
Suboptimal	39	14.00±3.39	7.37–20.64				
**Response to initial chemotherapy -no. (%)**				<0.0001	6.76	3.49–13.07	<0.0001
Sensitive	60	47.00±5.84	35.56–58.44				
Resistant or refractory	38	15.00±2.38	10.33–19.67				
**CA125 level (U/ml)**				0.917			
<600	43	31.00±4.95	21.29–40.71				
≥600	66	25.00±2.23	20.63–29.37				
**eIF4G1 expression**				0.026			
Low	31	41.00±10.00	21.40–60.60				
High	79	24.00±1.01	22.01–25.99				

* Log-rank test

# Cox regression test.

**Fig 6:**
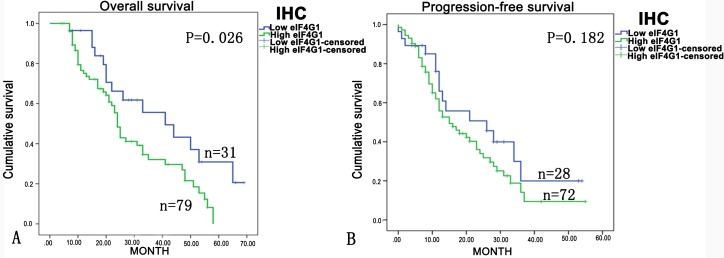
Post-surgical overall survival correlates with eIF4G1 protein expression in ovarian cancer. (A) Overall survival rates for cases with high eIF4G1 expression versus cases with low eIF4G1 expression levels in ovarian cancer patients. (B) Post-surgical progression free survival rates for cases with high eIF4G1 expression versus cases with low eIF4G1 expression levels in ovarian cancer patients. There were 40 patients with low eIF4G1 expression and 93 patients with high eIF4G1 expression. However, due to missing survival data, only 72 and 79 patients with high eIF4G1 as well as 28 and 31 patients with low eIF4G1 expression were included for progression-free survival analysis and overall survival analysis, respectively.

In order to determine whether there is any relationship between the level of eIF4G1 expression and the prognosis in ovarian cancer patients, we performed Kaplan–Meier analysis and the log-rank test. Patients who have lower eIF4G1 levels tend to experience a longer overall survival time (P = 0.026). However, the log-rank test showed that no difference in progression-free survival (P = 0.182) (Fig 6).

## Discussion

Ovarian cancer is a serious health problem for women because of its poor 5-year survival rate. Women diagnosed with early stage ovarian cancer (stages I to II) have five-year survival rates that range from 57% to 90%. By contrast, the five-year survival rates for patients who are diagnosed with advanced stage disease ovarian cancer range from 18% to 45% [14]. Two main factors account for this poor survival rate: late diagnosis and chemoresistance [15]. Therefore, discovery of accurate biomarkers associated with the diagnosis, prognosis, and/or treatment efficacy of ovarian cancer would benefit high-risk patients.

It is known that RNA-binding proteins are crucial regulatory proteins in cell biology and that these proteins regulate the stability, translocation, alternative splicing, and translational efficiency of RNAs [16–20]. Because of their critical roles in processes ranging from alternative splicing to RNA degradation, alterations in expression have been reported to be the cause of cancers such as hepatocellular carcinoma [21], colon cancer [22], prostate cancer [23] and melanoma [24]. Over the last decade, interest in RBP function in ovarian cancer is increasing.

eIF4G1, which is an RBP, serves as a scaffold protein that cooperates with cap-binding protein eIF4E and ATP-dependent RNA helicase eIF4A to locate the 5’ end of the mRNA, a key locus to reveal the initiation codon, and to facilitate mRNA recruitment to the ribosome [25]. eIF4G1 can bring the 5′ and 3′ ends of the mRNA together to form a 'closed-loop mRNP' by interacting with the other RBPs. Then the 'closed-loop mRNP' interacts with the pre-initiation complex to promote the coupling of translation termination and recycling events with subsequent rounds of initiation on the same mRNA [26]. Considering its crucial role in initiating cap-dependent translation, it has been reported that overexpression of eIF4G1 promotes both inflammatory breast cancer cell survival and the formation of tumor emboli [27]. In addition, studies have demonstrated that eIF4G1 promotes phenotypic responses that may assist tumor cells to develop drug resistance [28].

Thus, we hypothesized that altered expression of eIF4G1 plays a role in ovarian cancer. Using a series of fresh frozen human tissue specimens, we observed that eIF4G1 mRNA is up-regulated in ovarian cancer tissue compared to normal OSE specimens. Thus, eIF4G1 may act as a oncogene whose aberrant expression may be involved in tumorigenesis. To further investigate the relation between eIF4G1expression and the clinicopathological features in ovarian cancer, we followed 134 post-surgery patients. Furthermore, we performed IHC to examine the dynamics of eIF4G1 expression of different characters based on complete follow-up data in those 134 ovarian cancer tissues and in normal ovarian epithelial cell specimens. Our results demonstrated that the mean staining intensity of eIF4G1 in ovarian cancer tissues was significantly greater than the intensity in normal ovarian epithelial cell specimens, which is consistent with previous RT-PCR results. As mentioned above, the mRNA and protein expression levels of eIF4G1 in ovarian cancer tissues were remarkably higher than those in normal ovarian tissues, suggesting that the increased expression of eIF4G1 exists not only at the post-transcriptional level but also at the transcriptional level. Analyzing the correlation between eIF4G1 expression and clinicopathological features, we observed that eIF4G1 expression in early stages of ovarian cancer according to FIGO staging is significantly lower than in advanced stages. Thus, eIF4G1 may be a factor facilitating ovarian cancer. Moreover, our results also indicate that eIF4G1 expression correlates with the presence of omentum metastasis, supporting that this protein acts as an oncogene in the progression of ovarian cancer. Hence, eIF4G1 may provide a therapeutic target to impede abdominal metastasis.

To investigate the influence of eIF4G1 on the prognosis of ovarian cancer patients, we generated survival curves and compared the progression-free survival times and overall survival times according to the expression of eIF4G1 based on protein level. The results demonstrated that ovarian cancer patients with high expression of eIF4G1 tended to have lower overall survival rates. However, no significant differences were observed between the groups with the progression-free survival times. While checking TCGA Ovarian cohort (Cancer Genomics Browser) and analyzing the data of Agilent microarray, we found that the patients with high expression of eIF4G1 mRNA have a longer overall survival time (P = 0.004, S1 Fig). Then we assessed another public ovarian cohorts (kmplot.com) profiled on Affymetrix microarray for serous epithelial ovarian cancer with stage III or IV, and found that there is no statistical significance (S2 Fig). These microarray data seems not to be consistent with our finding. However, in checking with TCGA Ovarian cancer cohort (the cBioPortal for Cancer Genomics) (http://cbioportal.org) using RNA-seq data results in a P = 0.0448 for PFS and P = 0.0994 for OS (S3 Fig). These results are consistent with our findings. In addition, we also analyzed two public ovarian cancer microarrays (GSE18521 [12] and GSE40595 [13]) and the results demonstrated support that eIF4G1 is an oncogene. Further studies with larger sample sizes and longer follow-up times are required to confirm this association.

As mentioned above, previous studies have shown that eIF4G plays an essential role in the translation initiation [25–26]. There are two isoforms of eIF4G in mammals: eIF4G1 and eIF4G2. They have 46% identity at the amino acid level in humans. However, eIF4G1 is the prototype member of the family [29]. Central to the translation initiation is the translation initiation factor 4E (eIF4E), which recruits the small ribosomal subunit to the 5′ end of the mRNA through its interaction with the scaffold protein eIF4G. The eIF4E-binding protein (4E-BP) is a phosphorylation-dependent regulator of protein synthesis. The nonphosphorylated or minimally phosphorylated form of 4E-BP tightly binds and sequesters the eIF4E from binding to eIF4G and the recruitment of the small ribosomal subunit. Once phosphorylated by mammalian target of rapamycin complex 1(mTORC1), 4EBP dissociates from eIF4E, allowing eIF4E to interact with eIF4G and translation initiation to resume. The eIF4E/eIF4G interaction is highly regulated by competitive binding of 4EBPs, which are at a convergence point of signaling pathways and act as tumor suppressors. Hence, disrupting eIF4E binding to eIF4G provides an appealing strategy to control or treat cancer. A previous study has reported 4EBP-based eIF4E-binding peptides that prevent eIF4E from binding eIF4G, block cap-dependent translation, and inhibit cell growth in ovarian cancer cells [30]. Moreover, Naotaka Sekiyama and colleagues have discovered an eIF4E/eIF4G interaction inhibitor 1 (4EGI-1), which dissociates eIF4G from eIF4E but enhances 4EBP1 binding, demonstrating antitumor activity [31]. Thus, eIF4G1 can be a therapeutic target for ovarian cancer treatment.

In summary, our results exhibited the first evidence that eIF4G1 overexpression correlates with the development of ovarian cancer. This protein may be involved in the occurrence and progression of ovarian cancer and act as a player in the metastasis of ovarian cancer. Mechanistic studies remain to be undertaken to further unravel the role of eIF4G1 in ovarian cancer.

## Supporting Information

S1 Fig Overall survival rates for cases with high eIF4G1 expression versus cases with low eIF4G1 expression levels in 536 ovarian cancer patients from the TCGA cohort based on microarray(AgilentG4502A_07_3) (P = 0.004). (TIF)

S2 Fig Overall survival rates for cases with high eIF4G1 expression versus cases with low eIF4G1 expression levels checking with public ovarian cohorts (kmplot.com) profiled on Affymetrix microarray for stage 3+4 and serous histology. (A) Profiling on Affymetrix ID 208624_s_at; (B) Profiling on Affymetrix ID 208625_s_at.
(TIF)

S3 Fig Post-surgical progression free survival and overall survival of TCGA Ovarian cancer cohort (n = 307) (the cBioPortal for Cancer Genomics) according to eIF4G1 expression in cancer tissues (Log-rank test). (A) Post-surgical progression free survival rates for cases with high eIF4G1 expression versus cases with low eIF4G1 expression levels in ovarian cancer patients (P = 0.0448). (B) Overall survival rates for cases with high eIF4G1 expression versus cases with low eIF4G1 expression levels in ovarian cancer patients (P = 0.0994).
(TIF)

S1 Table The clinical data of the 134 patients with serous ovarian cancer. (XLSX)
