# Supplementary figures and images for: Characterization of the Expression of the RNA Binding Protein eIF4G1 and Its Clinicopathological Correlation with Serous Ovarian Cancer
Source: PLoS One. 2016 Sep 26;11(9):e0163447. doi: 10.1371/journal.pone.0163447 (PMC5036801; doi:10.1371/journal.pone.0163447)

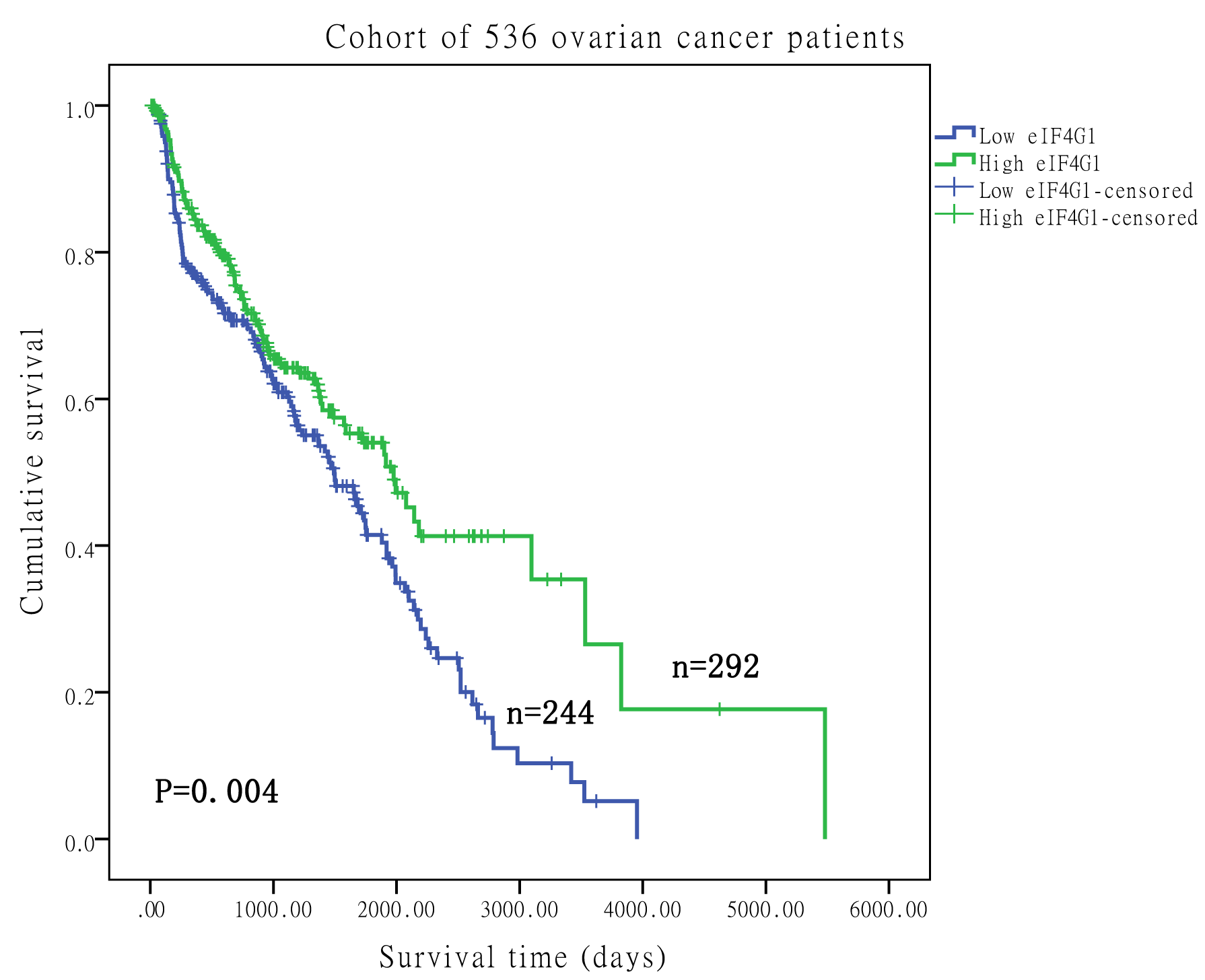

Supplement: S1 Fig — (TIF) [file pone.0163447.s001.tif]

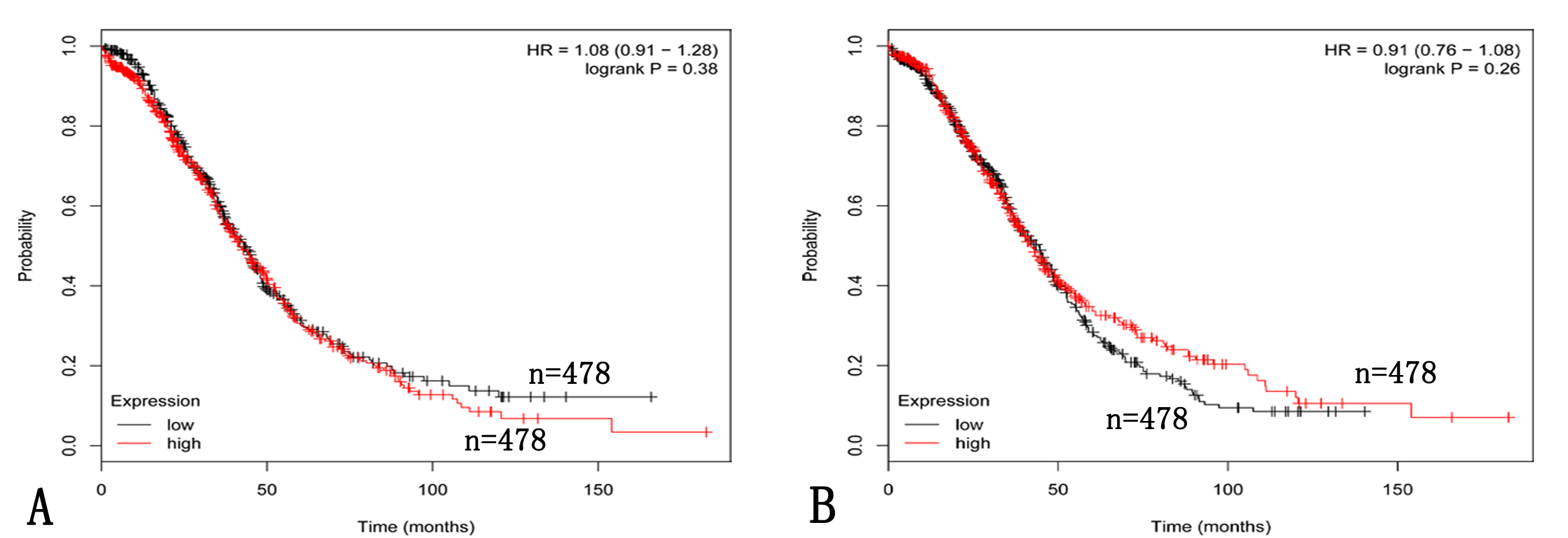

Supplement: S2 Fig — (A) Profiling on Affymetrix ID 208624_s_at; (B) Profiling on Affymetrix ID 208625_s_at. (TIF) [file pone.0163447.s002.tif]

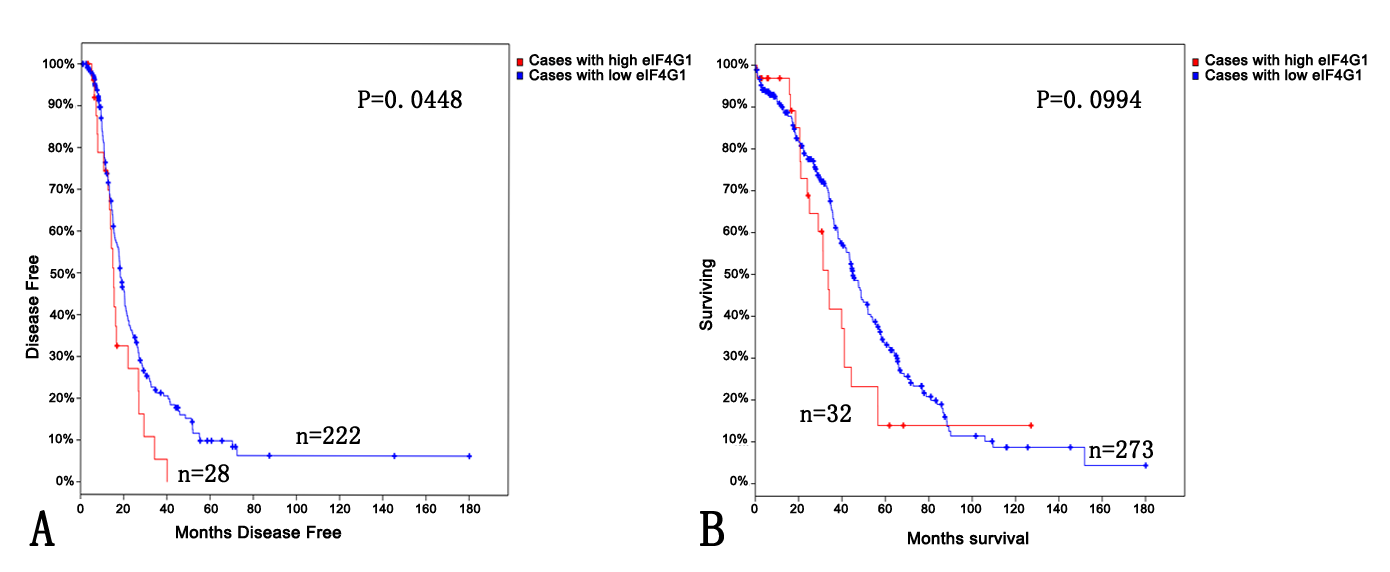

Supplement: S3 Fig — (A) Post-surgical progression free survival rates for cases with high eIF4G1 expression versus cases with low eIF4G1 expression levels in ovarian cancer patients (P = 0.0448). (B) Overall survival rates for cases with high eIF4G1 expression versus cases with low eIF4G1 expression levels in ovarian cancer patients (P = 0.0994). (TIF) [file pone.0163447.s003.tif]
